# An Unsupervised Deep Learning-Based Model Using Multiomics Data to Predict Prognosis of Patients with Stomach Adenocarcinoma

**DOI:** 10.1155/2022/5844846

**Published:** 2022-10-27

**Authors:** Sizhen Chen, Yiteng Zang, Biyun Xu, Beier Lu, Rongji Ma, Pengcheng Miao, Bingwei Chen

**Affiliations:** ^1^Department of Epidemiology and Biostatistics, School of Public Health, Southeast University, Nanjing 210009, China; ^2^Department of Biostatistics, Nanjing Drum Tower Hospital, The Affiliated Hospital of Nanjing University Medical School, Nanjing 210008, China

## Abstract

**Methods:**

Patients (363 in total) with stomach adenocarcinoma from The Cancer Genome Atlas (TCGA) cohort were included. An autoencoder was constructed to integrate the RNA sequencing, miRNA sequencing, and methylation data. The features of the bottleneck layer were used to perform the *k*-means clustering algorithm to obtain different subgroups for evaluating the prognosis-related risk of stomach adenocarcinoma. The model's robustness was verified using a 10-fold cross-validation (CV). Survival was analyzed by the Kaplan-Meier method. Univariate and multivariate Cox regression was used to estimate hazard risk. The model was validated in three independent cohorts with different endpoints.

**Results:**

The patients were divided into low-risk and high-risk groups according to the *k*-means clustering algorithm. The high-risk group had a significantly higher risk of poor survival (log-rank *P* value = 2.80*e* − 06; adjusted hazard ratio = 2.386, 95% confidence interval: 1.607~3.543), a concordance index (C-index) of 0.714, and a Brier score of 0.184. The model performed well both in the 10-fold CV procedure and three independent cohorts from the Gene Expression Omnibus (GEO) repository.

**Conclusions:**

A robust and generalizable model based on the autoencoder was proposed to integrate multiomics data and predict the prognosis of patients with stomach adenocarcinoma. The model demonstrates better performance than two alternative approaches on prognosis prediction. The results might provide the grounds for further exploring the potential biomarkers to predict the prognosis of patients with stomach adenocarcinoma.

## 1. Introduction

Stomach cancer is responsible for approximately 769,000 deaths in 2020 and is the fifth most common cancer and the fourth cause of global cancer-related mortality [1]. Stomach adenocarcinoma is the most prevalent histological subtype and has increasing incidence and mortality rates in recent years [2]. Compared to early diagnosis, the 5-year overall survival (OS) rate of stomach adenocarcinoma drops to less than 30% because of recurrence and metastasis at an advanced stage [3, 4]. Identification of the patients with high risk of stomach adenocarcinoma could help guide the future development of targeted treatment strategies and improve prognosis. Therefore, it is essential to develop a model for risk identification.

As the result of technological advancement and decreasing costs, high-throughput sequencing technology generated a large amount of multiomics data, which contains a wide spectrum of omics such as genome, transcriptome, and epigenome, and provides an opportunity to identify the risk of stomach adenocarcinoma at different molecular levels. Since single-omics data could only provide limited information, multiomics data could provide a holistic view of the biological system through identifying important biomarkers and providing biological information at multiple levels [5–7].Therefore, integration of multiomics data would provide better risk identification for patients with stomach adenocarcinoma.

Deep learning, also called deep neural network (DNN), is a new category of machine learning methods and widely used in many fields [8–11]. Deep learning consists of multiple layers containing multiple artificial neurons which have a weight and a shift value updated during backpropagation to minimize global loss function [12, 13]. An autoencoder is an unsupervised deep learning framework which is aimed at reconstructing its original input through a series of nonlinear transformations. The hidden layer could represent the information of the input layer. Compared to other methods for dimension reduction, the autoencoder is considered to fit a complex nonlinear relationship well. In recent years, many researchers paid attention to using autoencoder to integrate the information of multiomics and identifying the subtype of patients with cancer [14–16]. Some of the studies stacked the matrices of all multiomics data and then constructed one autoencoder to extract the latent information and to perform a clustering algorithm. Other studies constructed autoencoders for each type of the multiomics data and then stacked the nodes from each autoencoder for clustering.

In this study, an autoencoder was constructed to integrate multiomics data of patients from The Cancer Genome Atlas (TCGA) cohort with stomach adenocarcinoma to identify the risk and predict the prognosis, including mRNA expression, miRNA expression, and CpG methylation. The bottleneck layer was thought to represent the information of multiomics data, and then, the univariate Cox proportional hazard (Cox-PH) model was used to select the nodes in the bottleneck layer related to survival. The *k*-means clustering algorithm was performed to obtain the subgroup of patients with stomach adenocarcinoma. A Kaplan-Meier curve was drawn for survival analysis, and the log-rank method was used for statistical testing. The obtained subgroup was compared with two alternative approaches: principal component analysis (PCA) and similarity network fusion (SNF). A 10-fold cross-validation- (CV-) like was performed to assess the robustness, and three independent cohorts from the Gene Expression Omnibus (GEO) repository were used to validate the performance of the prognosis model.

## 2. Literature Review

Since more information could be provided by multiomics data compared to single-omics data, many algorithms were proposed to integrate different types of omics data, such as similarity network fusion (SNF) [17] and iCluster [18]. This section highlights the studies identifying subgroups of patients with cancer using a deep learning-based model to predict the prognosis and identify potential prognostic biomarkers.  Table 1 presents the review on applications of an unsupervised deep learning model in subgroup identification and prognosis prediction for patients with cancer.

To compare the performance of different deep learning autoencoders for cancer subtype identification using multiomics data, Franco et al. [27] performed four autoencoders (vanilla, denoising, sparse, and variational) on four types of cancer (glioblastoma multiforme, GBM; colon adenocarcinoma, COAD; kidney renal clear cell carcinoma, KRCC; breast invasive carcinoma, BIC) from TCGA datasets. The study concluded that generally vanilla and variational autoencoders showed the best performance to identify different subgroups, in spite of the varied performance of different autoencoders on different datasets.

Besides, some deep learning frameworks were proposed to integrate multiomics data of patients with cancer to identify the subgroup and predict the prognosis. A framework to integrate multiomics data by a denoising autoencoder for accurate cancer prognosis prediction (DCAP) was proposed by Chai et al. [28]. The framework was applied in 15 TCGA datasets and presented superior accuracy and robustness.

Poirion et al. [29] proposed an ensemble framework of deep learning and machine learning approaches, named DeepProg, to robustly predict the survival subgroups of patients. DeepProg presents superior predictive accuracy and robustness on 32 cancers from TCGA datasets.

Yang et al. [30] proposed Subtype-GAN, which uses a generative adversarial network (GAN) to extract latent variables and uses consensus clustering and the Gaussian Mixture model to identify tumor samples' subgroups. The result proved the well performance in ten TCGA datasets.

Some of the frameworks involve other types of information beside multiomics data to obtain the subgroup and predict the prognosis. Liu et al. [31] proposed deep learning fusion clustering (DLFS) to integrate multiomics data on TCGA-BCRA dataset for breast cancer subgroup detection. DLFS involves both clustering loss and classification loss into the total loss of the whole framework. Classification loss is calculated based on prior biological knowledge.

Zhao et al. [32] proposed a scalable and interpretable multiomics deep learning framework named DeepOmix to identify the relationships between multiomics data and the survival data and to incorporate some prior biological information. The output layer of DeepOmix consists of survival time and status, and patients would be divided into high- and low-risk subgroups based on the output layer. More and more types of data like single-cell RNA sequencing data or spatial transcriptomics data would be available for the DeepOmix framework in the future.

However, only few studies constructed a deep learning-based model for detecting the subgroups of patients with stomach adenocarcinoma to predict the prognosis and identify the prognostic biomarkers.

## 3. Materials and Methods

### 3.1. Data Collection and Preprocessing

In this study, TCGA-Assembler package [33] in R was used to derive the multiomics data of 363 patients with stomach adenocarcinoma from the TCGA cohort, including RNA sequencing (RNA-Seq) data (UNC IlluminaHiSeq_RNASeqV2; Level 3), microRNA sequencing (miRNA-Seq) data (BCGSC IlluminaHiSeq_miRNASeq; Level 3), and DNA methylation data (JHU-USC HumanMethylation450; Level 3). For mRNA and miRNA data, features which have zero value in more than 20% samples were excluded. And then, samples which have zero value in more than 20% features were excluded. For DNA methylation data, average methylation *β*-value of all CpG within the gene promoter region, which is defined as 1500 base pairs (bp) upstream of transcription start sites (TSS) of genes, was calculated as the promoter methylation values. Promoter features which have missing value in more than 20% samples were excluded, and samples which have missing value in more than 20% promoter features were excluded. And then, the missing values were imputed by the impute package (https://bioconductor.org/packages/release/bioc/html/impute.html) in R. All the features obtained above were unit scaled by samples as follows:

(1) $$\begin{array}{c} v_{normed}=v\frac{1}{{\left\|v\right\|}_{2}^{2}}, \end{array}$$

where *v* is a vector of samples and ‖*v*‖_2_^2^ is the *l*_2_ norm of *v*.

To validate the performance, three independent cohorts obtained from the GEO dataset were used. The first cohort was obtained from GSE15459 [34–39], which derived the RNA-Seq data of 192 samples using the Affymetrix GeneChip Human Genome U133 Plus 2.0 Array. The patients of GSE15459 were recruited from the National Cancer Center and hospitals of the National Healthcare Group. The second cohort was obtained from GSE26253 [40, 41] Illumina HumanRef-8 WG-DASL v3.0 dataset with 432 samples. Samples of the GSE26253 cohort were from Samsung Medical Center and passed the RNA quality control. The third cohort was obtained from the GSE84437 [42] Illumina HumanHT-12 V3.0 expression beadchip microarray dataset with 433 samples. Total RNA of samples from GSE84437 was extracted from the fresh-frozen gastrectomy specimens at the Yonsei University Severance Hospital (South Korea) between 2000 and 2010. According to the annotation file from each platform, average expression of all probes corresponding to one gene symbol was calculated as the mRNA expression value. Unit scaling was also performed on each cohort. OS, disease-free survival (DFS), and the response of fourfold increase from the baseline of the heterotopic tumor size were used as the endpoint of the three cohorts.

### 3.2. Autoencoder Construction

Autoencoder is an unsupervised feedforward neural network. According to the previous study [14], more hidden layers contribute little to the final performance but lead to a large amount of calculation. Thus, an autoencoder with three hidden layers was implemented using the Python library Keras. The number of nodes of hidden layers was set to 2000, 500, and 2000 (Supplementary Table S1). Though the model with 5000, 500, and 5000 nodes in each hidden layer provided the lowest log-rank *P* value and the highest concordance index (C-index), the model was not chosen because of its bad performance in the 10-fold CV procedure (Supplementary Table S2). The layer with 500 nodes named the bottleneck layer was thought to contain the representation features. Autoencoder reconstructs its input layer *x* by a series of nonlinear transformations. In this study, tanh was used as the activation function of each layer. A loss function of the mean square error was used to measure the error between input layer *x* and output layer *x*′: *L*(*x*, *x*′) = (*x* − *x*′)^2^. However, to avoid overfitting, a batch normalization layer was appended after the hidden layer. Moreover, an adaptive momentum (Adam) algorithm was used with 32 epochs.

### 3.3. Selection of Representation Features and *k*-Means Clustering

For each representation feature obtained from the bottleneck layer, a univariate Cox proportional hazards (Cox-PH) model was built using survival package in R, and the features with *P* value greater than 0.10 were screened out. The *k*-means clustering algorithm was applied to the remaining features. Silhouette index [43] and Calinski-Harabasz criterion [44] were calculated to determine the optimal number of clusters, and the label of each patient was obtained. On account of the labels being related to the prognosis risk of stomach adenocarcinoma, we used “subgroup” instead of label or cluster. Kaplan-Meier survival curves of different subgroups were drawn, and log-rank *P* value, C-index, and Brier score were calculated.

### 3.4. Supervised Classification

To verify the robustness of the subgroup obtained from the *k*-means algorithm, internal and an external validations were performed.

In order to ensure sufficient sample size of the test set, a 10-fold CV-like procedure [14, 15, 21] was used to partition the TCGA dataset into a training set and a test set in the internal validation. Through the 10-fold CV-like procedure, 363 samples were randomly split into 5 folds, and then, ten different combinations of 3 folds (training sets) and 2 folds (test sets) were obtained. For each of these ten combinations, a different autoencoder was built using the data from the training set, and *k*-means clustering was performed to obtain the subgroup labels. Then, the top 50 mRNA, 30 miRNA, and 50 methylation features were selected based on the *P* value of analysis of variance (ANOVA) to build a support vector machine (SVM) model. Three single-omics data and the multiomics data were used to build a SVM model, respectively. Subgroup labels of samples from the test sets were predicted by the SVM classifier. Then, log-rank *P* value, C-index, and Brier score were calculated to evaluate the robustness of the prognosis model.

In the external validation, the mRNA features within both the TCGA cohort and the independent cohort were firstly selected. Then, like in the internal validation, a SVM classifier was built using the top 50 mRNA features, which were selected by the *P* value of ANOVA, of the whole TCGA cohort. The subgroup labels of three independent cohorts were predicted, and the log-rank *P* value, C-index, and Brier score were calculated.

However, two scaling steps were applied before constructing a SVM classifier whether in training sets, test sets, or the independent cohort. For the mRNA and methylation features, median scale normalization and robust scale normalization were performed. And for miRNA features, median scale normalization and unit scale normalization were performed. Unit scale normalization was shown in the aforementioned procedure, and the median and robust scale normalization were performed as follows:

(2) $$\begin{array}{c} \text{median scale normalization}:x_{\text{scaled}}=\frac{\left(x-\text{median}\left(x\right)\right)}{\text{mad}\left(x\right)},\\ \text{robust scale normalization}:x_{\text{whitened}}=\left\{\frac{x_{i}-{\text{mean}}_{25-75}\left(x_{i}\right)}{{sd}_{25-75}\left(x_{i}\right)},x_{i}\in x\right\}, \end{array}$$

where *x* = (*x*_1_, ⋯, *x*_*n*_) is a vector of feature and mad(*x*) = median({|*x*_*i*_ − median(*x*)|, *x*_*i*_ ∈ *x*}).

All SVM classifiers were constructed using the e1071 package (https://CRAN.R-project.org/package=e1071) in R, and 5-fold CV was used to perform a grid search of the best hyperparameters. C-index and Brier score were calculated using the survcomp package [45] in R.

### 3.5. Alternative Approaches

Two alternative approaches were used to compare with the autoencoder. In the first approach, principal component analysis (PCA) was performed and the number of principal components was set to the same as the bottleneck layer. Then, a univariate Cox-PH model was built for each principal component, and the *k*-means clustering was performed to obtain the subgroups which indicated to the prognosis-related risk. In the second approach, similarity network fusion (SNF) [17] was used to construct sample similarity matrices for each of the omics data types using pairwise correlation. Through the fused network of samples, the optimal numbers and subgroup labels were obtained according to the spectral clustering algorithm. The SNF was implemented using the SNFtool package in R.

### 3.6. Bioinformatics Analysis

Differential expression analysis was performed between different subgroups of the TCGA cohort. For mRNA and miRNA data, the DESeq2 package in R was used to identify the differentially expressed genes (DEGs) and miRNA expression. The features of the mRNA or miRNA which satisfy the criteria of |log2FC| > 1 and false discovery rate (FDR) < 0.05 were considered to be significant. For methylation data, beta value was transformed into *M* value using the lumi package [46] in R, and then, significant differentially methylated genes (DMGs) were identified in the criteria of |*M* value difference| > 1 and FDR < 0.05 using moderate *t*-test with the limma package [47] in R.

Kyoto Encyclopedia of Genes and Genomes (KEGG) enriched analysis was performed for the significant DEGs, which were obtained from the differential expression analysis, with the threshold of FDR < 0.05, using the clusterProfiler package [48, 49] in R.

## 4. Results

### 4.1. Two Significant Subgroups Were Identified according to the TCGA Cohort

**Figure 1:**
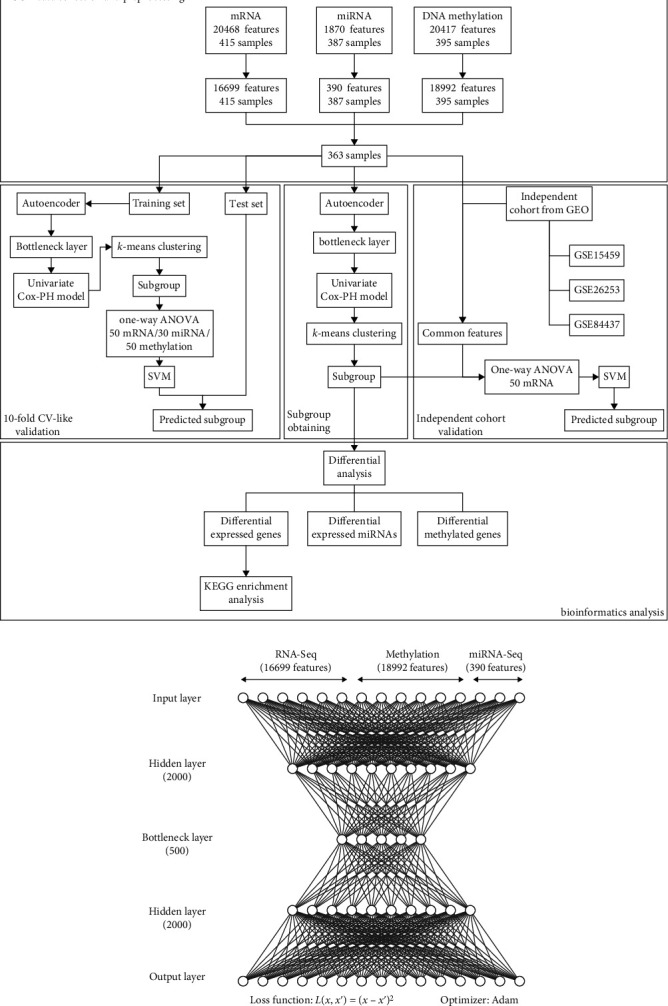
Architecture of autoencoder and overall workflow. (a) The workflow of overall study. (b) The architecture of autoencoder to integrate multiomics data.

The workflow of overall study is shown in Figure 1(a). From the TCGA-STAD project, the total number of patients with stomach adenocarcinoma included in this study was 363. The clinical information of the TCGA cohort is shown in Table 2. After preprocessing, 16,699 genes from RNA-Seq, 390 miRNAs from miRNA-Seq, and 18,992 genes from DNA methylation data were obtained as input features. Three omics data were stacked as the features of the input layer. The architecture of the autoencoder is shown in Figure 1(b).

**Figure 2:**
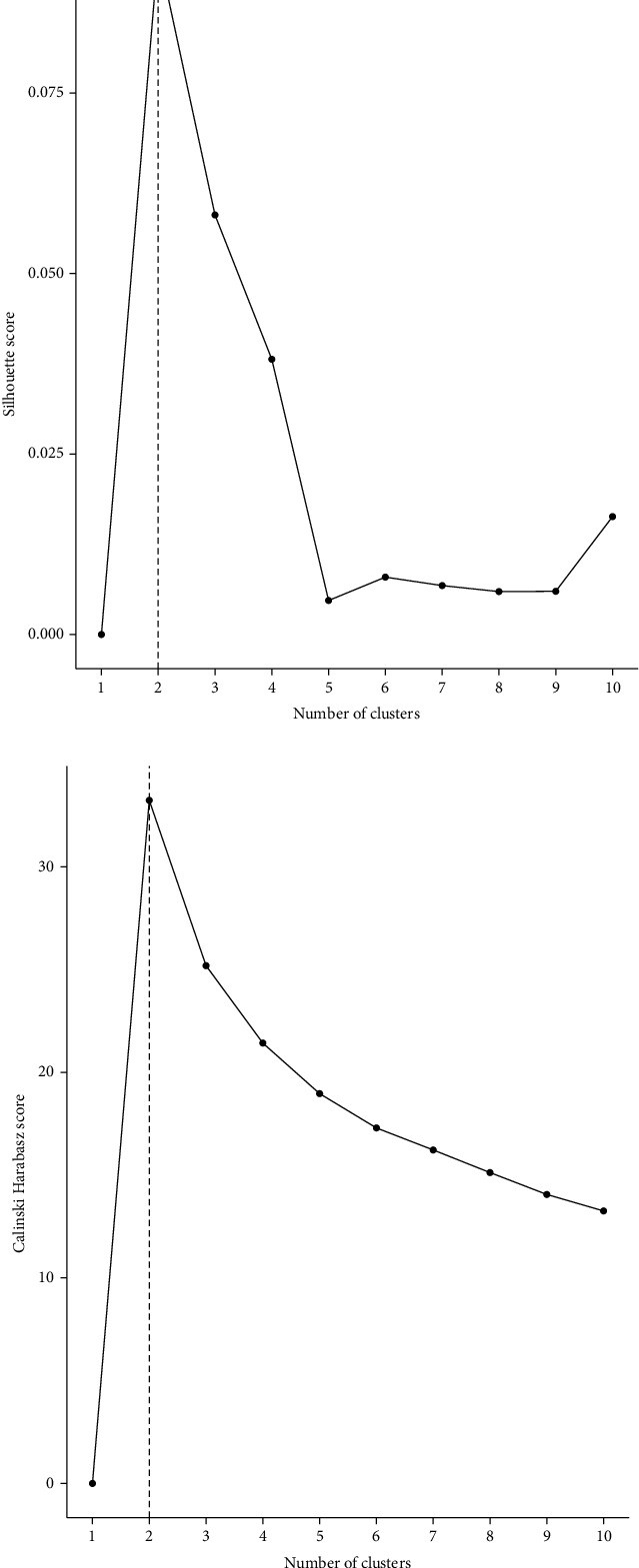
Selection of the best cluster *K* according to silhouette index and Calinski-Harabasz score: (a) silhouette index; (b) Calinski-Harabasz score.

The representation features obtained from the bottleneck layer were selected using the univariate COX-PH model, and the cut-off of *P* value was set to 0.10. Then, 104 features were thought to be related to survival and used to implement the *k*-means clustering. According to the silhouette index and the Calinski-Harabasz criterion, *K* = 2 could be considered as the optimal number of clusters (Figure 2). Thus, two subgroups were obtained (Supplementary Table S3).

**Figure 3:**
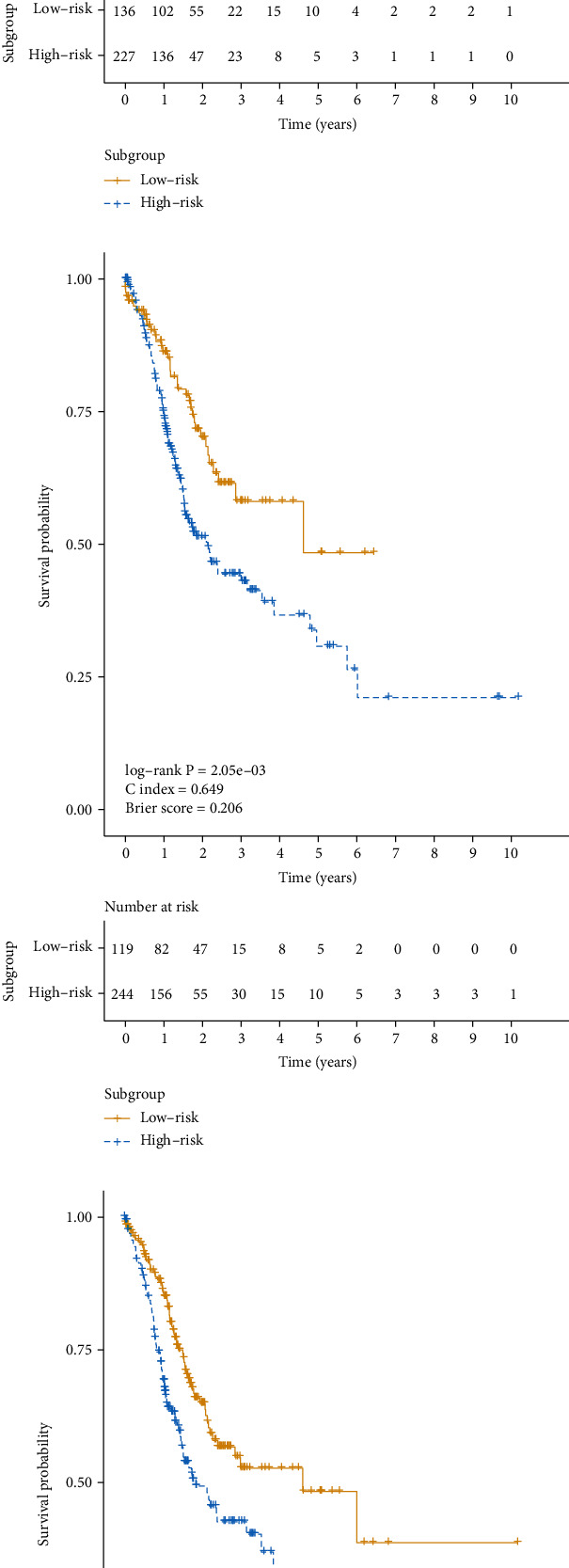
Kaplan–Meier curves for OS time between two different subgroups of TCGA cohort obtained from different approaches: (a) autoencoder; (b) PCA; (c) SNF.

Furthermore, prognostic difference between these two subgroups was assessed using the Cox-PH model. The log-rank *P* value, C-index, and Brier score were 2.8*e* − 06, 0.714, and 0.184, respectively (Figure 3(a)). However, 104 features were obtained after a dimension reduction method, PCA, and *k*-means clustering were performed. The log-rank *P* value, C-index, and Brier score were 2.05*e* − 03, 0.649, and 0.206, respectively (Figure 3(b)). And a SNF was constructed with number of neighbors *K*, hyperparameter sigma, and number of iteration T setting to 14, 0.3, and 27, respectively. The log-rank *P* value, C-index, and Brier score were 8.91*e* − 04, 0.653, and 0.200, respectively (Figure 3(c)). Thus, the subgroup obtained from the autoencoder performed better than those from two other alternative approaches.

### 4.2. Cox-PH Model including Subgroups and Other Clinical Features

As the univariate Cox-PH models (Table 3) showed, subgroup (log-rank *P* value 2.81*e* − 06), age (log-rank *P* value 1.08*e* − 02), pathologic tumor (T) stage (log-rank *P* value 3.23*e* − 02), pathologic node (N) stage (log-rank *P* value 5.95*e* − 03), and stage (log-rank *P* value 3.20*e* − 03) were identified as risk factors related to OS time. The multivariate Cox regression model also found the same result (adjusted HR 2.386; 95% CI 1.607-3.543; *P* value 1.62*e* − 05) after the adjustment of age, pathologic T stage, pathologic N stage, and stage.

### 4.3. Internal Validation to Assess the Robustness of the Subgroups

Since the two subgroups of the TCGA cohort were obtained from *k*-means clustering, a 10-fold CV-like procedure was performed to assess the robustness. In each fold, ANOVA was performed to select the top 50 mRNA, 30 miRNA, and 50 DNA methylation gene features associated with the obtained subgroup (Supplementary Table 4). The remaining features were used to build a SVM classifier which used the subgroup obtained from the autoencoder as the true label. For the training set, a C-index of 0.677 ± 0.042, a low Brier score of 0.216 ± 0.009, and a significant log-rank *P* value of 3.51*e* − 03 were generated (Table 4). For the test set, a C-index of 0.644 ± 0.030, a low Brier score of 0.209 ± 0.006, and a significant log-rank *P* value of 3.19*e* − 03 were generated (Table 4). SVMs using each three-single omics features were also built, and impressive performances were also produced. For the test set based on mRNA features only, a C-index of 0.636 ± 0.020, a Brier score of 0.208 ± 0.008, and a log-rank *P* value of 3.56*e* − 03 were generated (Table 4). For the test set based on miRNA features only, a C-index of 0.631 ± 0.017, a Brier score of 0.201 ± 0.006, and a log-rank *P* value of 1.81*e* − 03 were generated (Table 4). For the test set based on methylation features only, a C-index of 0.653 ± 0.016, a Brier score of 0.213 ± 0.004, and a log-rank *P* value of 2.63*e* − 03 were generated (Table 4).

### 4.4. External Validation in Three Independent Cohorts

**Figure 4:**
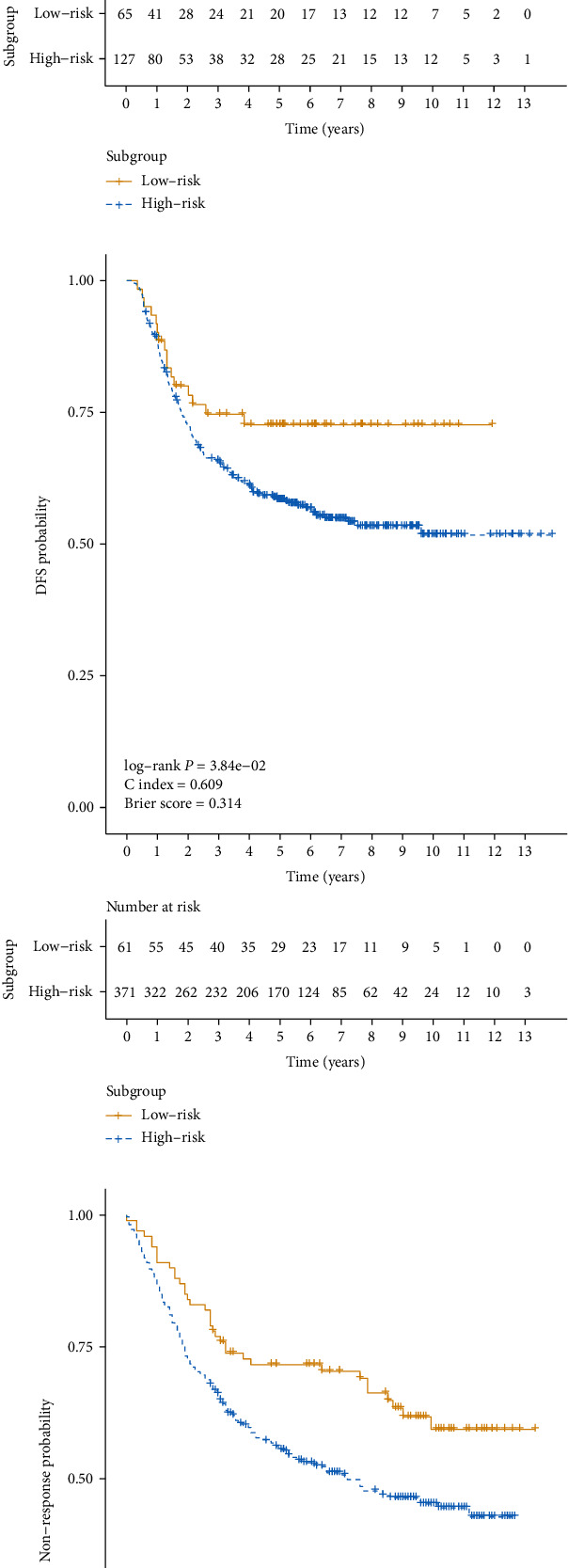
Kaplan–Meier curves between two different subgroups of three independent cohorts: (a) GSE15459; (b) GSE26253; (c) GSE84437.

To prove the performance of the classification model at predicting prognosis outcomes, three independent cohorts, GSE15459, GSE26253, and GSE84437, were used. The clinical information of the three cohorts is shown in Table 2. 14,881, 14,920, and 10,973 common mRNA features were used to select the top 50 mRNA features associated with the subgroup obtained from the prognosis model based on the autoencoder for the three independent cohorts, respectively. The GSE15459 cohort produced a C-index of 0.615, a Brier score of 0.219, and a significant log-rank *P* value of 1.19*e* − 02 (Figure 4(a)). The GSE26253 cohort produced a C-index of 0.609, a Brier score of 0.314, and a significant log-rank *P* value of 3.84*e* − 02 (Figure 4(b)). The GSE84437 cohort produced a C-index of 0.635, a Brier score of 0.278, and a significant log-rank *P* value of 4.10*e* − 03 (Figure 4(c)).

### 4.5. Bioinformatics Analysis

**Figure 5:**
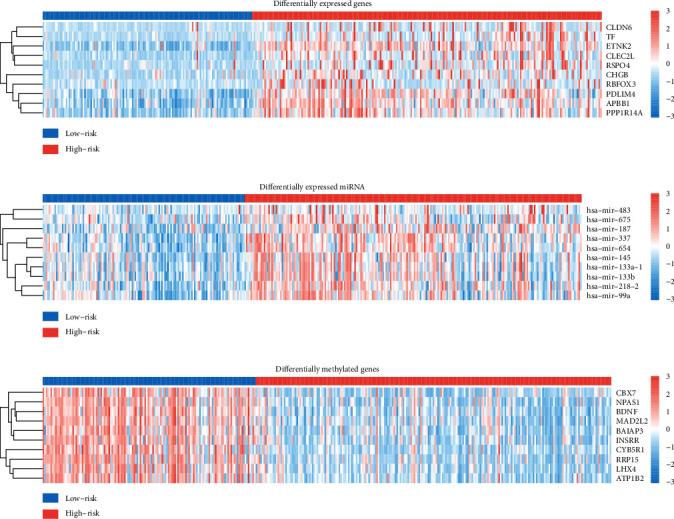
Heat map for the top 10 DEGs, differentially expressed miRNAs, and DMGs: (a) DEGs; (b) differentially expressed miRNAs; (c) DMGs.

Differential analysis was performed to detect the differentially expressed genes (DEGs), differentially expressed miRNAs, and differentially methylated genes (DMGs). 1839 upregulated and 56 downregulated genes were obtained with a criterion of |log2FC| < 1 and FDR < 0.05. 27 differentially expressed miRNA were obtained with the same criterion as DEGs. 1323 DMGs were obtained with a criterion of |*M* value difference| > 1 and FDR < 0.05. The top 10 DEGs (CLDN6, TF, ETNK2, CLEC2L, RSPO4, CHGB, RBFOX3, PDLIM4, APBB1, PPP1R14A), differentially expressed miRNAs (hsa-mir-483, hsa-mir-675, hsa-mir-187, hsa-mir-337, hsa-mir-654, hsa-mir-145, hsa-mir-133a-1, hsa-mir-133b, hsa-mir-218-2, hsa-mir-99a), and DMGs (CBX7, NPAS1, BDNF, MAD2L2, BAIAP3, INSRR, CYB5R1, RRP15, LHX4, ATP1B2) are shown in Figure 5.

**Figure 6:**
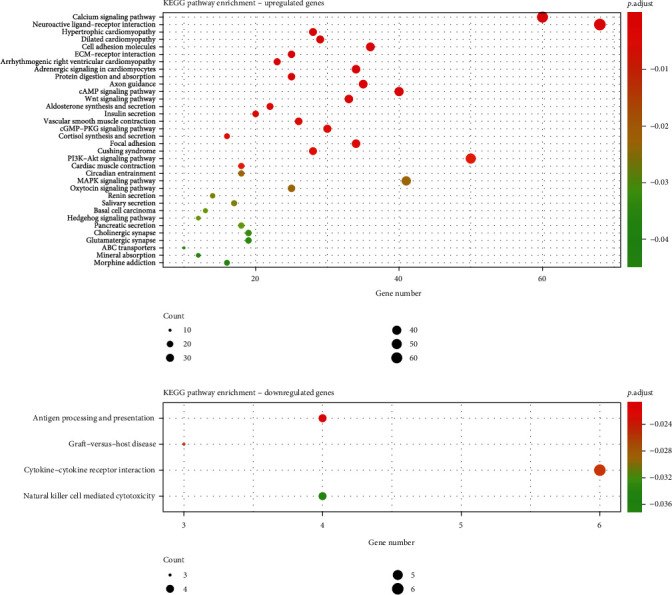
KEGG pathway enrichment analysis.

As the result of implementation of KEGG pathway enrichment analysis, the upregulated and downregulated DEGs were significantly involved in 30 and 8 signaling pathways, respectively (Figure 6 and Supplementary Table S5).

## 5. Discussion

Wide popularity and poor prognosis of stomach adenocarcinoma make it an urgent request for risk identification. In previous studies, deep learning-based unsupervised methods were implemented using multiomics data and obtained better prognosis performances [14–16]. The autoencoder is an architecture of deep learning which is able to fit a complex nonlinear relationship between the input layer and the output layer. The representative features from the bottleneck layer could reflect the information of multiomics data well, and the deep learning-based model overcomes the problem of high dimension of the multiomics data. In this study, two subgroups were identified using an autoencoder deep learning architecture. Difference of survival probabilities between two subgroups was significant, and the model fitness performed well. Performance of the model based on the autoencoder was better than those of two alternative approaches, PCA and SNF. It suggested that a deep learning-based method like autoencoder could utilize the information of multiomics data better through capturing the nonlinear relationship than the traditional dimension reduction method like PCA, and the model based on an autoencoder could produce a more accurate prediction of prognosis than the model which constructs and fuses a similarity network to have a comprehensive view of a biological process in specific diseases like SNF. The deep learning-based model would contribute to predicting the prognosis of patients with stomach adenocarcinoma and identifying the potential prognostic biomarkers.

According to the 10-fold CV-like validation, the robustness of the model identifying the subgroups has been verified. In addition, the subgroup could be considered as a risk factor according to the univariate and multivariate Cox-PH model. Moreover, the model was validated in three independent cohorts with different endpoints, and each cohort produced a significant log-rank *P* value. Regretfully, GSE26253 and GSE84437, with an endpoint of DFS and response of fourfold increasement of tumor size, respectively, produced a high Brier score, indicating high error of the model fitting on survival data. This suggests that the endpoint of OS would be more suitable in prognosis prediction using autoencoder-integrating multiomics data.

In fact, Xu et al. [26] have already used a deep learning architecture of bidirectional deep neural networks (BiDNNs) to integrate RNA-Seq and DNA methylation data of patients with gastric cancer to obtain two subgroups associated with prognosis-related risk. The model based on the autoencoder shows a better performance than that on BiDNNs, in log-rank *P* value (2.80*e* − 06 vs. 9.05*e* − 05, respectively) and C-index (0.714 vs. 0.673, respectively). This may indicate that complex architecture could not provide a better performance. Information from miRNA expression may also play an important role in the model based on the autoencoder to show its robustness. Both studies provide guidance for personalization of medical services.

DEGs, differentially expressed miRNAs, and DMGs between two subgroups were obtained and provided an opportunity to be used as potential prognostic biomarkers or candidate therapeutic targets in future clinical practice. Differential analysis and enrichment analysis were practiced to understand the difference between two subgroups at the molecular level. Some studies have revealed the relationship between the top 10 DEGs, DMGs, and stomach adenocarcinoma. Expression of CLDN6 indicates poor prognosis of gastric cancer [50–52]. RBFOX3 was considered to be associated to the growth and progression of gastric cancer [53]. CBX7 was thought to be related to the proliferation and metastasis of the gastric cancer cell and be able to reveal the prognosis [54–56]. BDNF expression was reported to be associated with poor prognosis of gastric cancer [57, 58]. CLDN6, TF, CLEC2L, and APBB1 were also identified as top 10 DEGs, and no common top 10 DMGs were found in the previous study using the model based on BiDNNs [26]. Low has-miR-337-3p expression was reported to be related to lymph node metastasis of human gastric cancer [59]. hsa-miR-145 and hsa-mir-133a-1 were considered to serve as an underlying prognostic indicator for the patient with stomach adenocarcinoma [60]. Little was known about the relationship between the other 7 of the top 10 differentially expressed miRNAs and stomach adenocarcinomas.

However, only RNA-Seq, miRNA-Seq, and DNA methylation data were used in this study. This might not provide sufficient information of the biological process, and more omics data could be included in the further study. Regretfully, no independent cohort with miRNA-Seq and DNA methylation data has been found to use in the external validation. Moreover, the poor interpretability of deep learning may prevent the prognosis model from being widely used in clinical practice, and a more theoretic approach is required to explain how the model works.

## 6. Conclusions

Prognosis-related risk was evaluated based on the architecture of the autoencoder, and the patients were divided into low-risk and high-risk groups. The high-risk group had a significantly higher risk of poor survival with an adjusted HR of 2.386, a log-rank *P* value of 2.80*e* − 06, a C-index of 0.714, and a Brier score of 0.184. The robustness of the prognosis model was successfully validated using a 10-fold CV-like procedure, and the performance was verified in three independent cohorts. The model based on the autoencoder has a better performance than PCA (log-rank *P* value: 2.05*e* − 03; C-index: 0.649) and SNF (log-rank *P* value: 8.91*e* − 03; C-index: 0.653) on prognosis prediction. DEGs, differentially expressed miRNAs, and DMGs between two subgroups were uncovered. The results might provide the foundation for further exploring the potential biomarkers to predict the prognosis of patients with stomach adenocarcinoma.

## Figures and Tables

**Table 1:** Deep learning applications in subgroup identification and prognosis prediction for patients with cancer.

Ref.	Datasets	Omics data	Deep learning model	Validation cohort
[14]	TCGA-HCC	mRNA miRNA DNA methylation	Autoencoder	LIRI-JP cohort NCI cohort miRNA GSE31384 cohort E-TABM-36 cohort Hawaiian cohort

[15]	TCGA-ESCC	mRNA DNA methylation	Autoencoder with early-fusion strategy and joint multimodal representation strategy	E-GEOD-53624 E-GEOD-53624

[16]	TCGA-HNSCC	mRNA miRNA DNA methylation	Autoencoder	E-GEOD-26549 E-GEOD-27020 E-GEOD-65858

[19]	TCGA-PRAD	mRNA miRNA DNA methylation	Autoencoder	—

[20]	TCGA-BLCA	mRNA miRNA DNA methylation	Autoencoder	GSE84525

[21]	TCGA-COAD	mRNA miRNA DNA methylation	Autoencoder	E-GEOD-17538 E-GEOD-39582 E-GEOD-28722

[22]	TCGA-BRCA	mRNA CNV	Autoencoder	—

[23]	TCGA-LUAD	mRNA miRNA DNA methylation CNV	Autoencoder	GSE81089 GSE63805 GSE63384 TCGA-LUAD CNV (lack of mRNA, miRNA, or methylation information)

[24]	TCGA-OV	mRNA/RNA-seq, CNV, DNA methylation	VAE, MMD-VAE	—

[25]	TCGA-PAAD	mRNA miRNA DNA methylation	Autoencoder	—

[26]	TCGA-STAD	mRNA DNA methylation	BiDNN	E-GEOD-62254 E-GEOD-26253

CNV: copy number variation; VAE: variational autoencoder; MMD-VAE: maximum mean discrepancy variational autoencoder; BiDNN: bidirectional deep neural network.

**Table 2:** Clinical information of TCGA and three independent cohorts.

Item	TCGA (*n* = 363)	GSE15459 (*n* = 192)	GSE26253 (*n* = 432)	GSE 84437 (*n* = 433)
Age (years; mean ± sd)	64.98 ± 10.55	64.37 ± 13.24		60.06 ± 11.58
Gender (female/male)	125/238	67/125		137/296
Race (Asian/Black or African American/White/not reported)	84/12/236/31			
Time (days; median)	463	574	1735	2087
Status (0/1)^a^	219/144	97/95	255/177	224/209
Stage (I/II/III/IV/-)	46/118/161/28/10	31/29/72/60/0	68/167/130/67/0	
Pathologic T stage (T1/T2/T3/T4)	18/72/168/105			11/38/92/292
Pathologic N stage (N0/N1/N2/N3/NX/-)	112/95/72/76/6/2			80/188/132/33/0/0
Pathologic M stage (M0/M1/MX)	327/19/17			

^a^Status 1 means reaching the endpoint. For TCGA and GSE15459, the endpoint is OS. For GSE26253, the endpoint is DFS. For GSE84437, the endpoint is the response of the fourfold increase from the baseline of the heterotopic tumor size.

**Table 3:** Identification for risk factor using univariate and multivariate Cox-PH model.

Factors	Univariate				Multivariate			
	HR	95% CI	*z*	*P* value^a^	HR	95% CI	*z*	*P* value^b^
Subgroup				2.81*e* − 06				
Low risk	1.000	—	—	—	1.000	—	—	—
High risk	2.392	(1.642, 3.485)	4.545	5.51*e* − 06	2.386	(1.607, 3.543)	4.312	1.62*e* − 05
Age				1.08*e* − 02				
<65	1.000	—	—	—	1.000	—	—	—
≥65	1.538	(1.102, 2.148)	2.528	1.15*e* − 02	1.982	(1.389, 2.829)	3.769	1.64*e* − 04
Gender				7.59*e* − 02				
Female	1.000	—	—	—				
Male	1.386	(0.965, 1.992)	1.767	7.72*e* − 02				
Race				5.22*e* − 01				
Not reported	1.000	—	—	—				
Asian	0.812	(0.430, 1.534)	-0.641	5.21*e* − 01				
Black or African American	1.478	(0.636, 3.434)	0.907	3.64*e* − 01				
White	0.919	(0.544, 1.552)	-0.316	7.52*e* − 01				
Pathologic T stage				3.23*e* − 02				
T1	1.000	—	—	—	1.000	—	—	—
T2	3.032	(0.716, 12.845)	1.506	1.32*e* − 01	1.935	(0.433, 8.637)	0.864	3.87*e* − 01
T3	4.457	(1.093, 18.180)	2.083	3.72*e* − 02	4.233	(0.846, 21.18)	1.756	7.90*e* − 02
T4	4.902	(1.188, 20.231)	2.198	2.80*e* − 02	4.738	(0.93, 24.147)	1.872	6.12*e* − 02
Pathologic N stage				5.95*e* − 03				
N0	1.000	—	—	—	1.000	—	—	—
N1	1.560	(0.973, 2.50)	1.847	6.47*e* − 02	1.583	(0.794, 3.154)	1.305	1.92*e* − 01
N2	1.628	(0.976, 2.71)	1.868	6.17*e* − 02	1.646	(0.718, 3.771)	1.178	2.39*e* − 01
N3	2.430	(1.520, 3.890)	3.708	2.09*e* − 04	2.390	(1.041, 5.485)	2.055	3.99*e* − 02
NX or missing	1.812	(0.432, 7.600)	0.812	4.17*e* − 01	1.566	(0.352, 6.970)	0.588	5.56*e* − 01
Pathologic M stage				1.04*e* − 01				
M0	1.000	—	—	—				
M1	1.785	(0.936, 3.410)	1.759	7.86*e* − 02				
MX	1.627	(0.759, 3.490)	1.251	2.11*e* − 01				
Stage				3.20*e* − 03				
I	1.000	—	—	—	1.000	—	—	—
II	1.362	(0.706, 2.630)	0.923	3.56*e* − 01	0.586	(0.221, 1.556)	-1.072	2.84*e* − 01
III	2.084	(1.130, 3.850)	2.350	1.88*e* − 02	0.518	(0.142, 1.892)	-0.996	3.19*e* − 01
IV	2.946	(1.406, 6.170)	2.863	4.20*e* − 03	0.842	(0.232, 3.057)	-0.262	7.93*e* − 01
Missing	3.760	(1.319, 10.72)	2.479	1.32*e* − 02	1.457	(0.349, 6.082)	0.516	6.06*e* − 01

^a^Both log-rank *P* value of each univariate Cox-PH model and *P* value of each variable were calculated. ^b^Score test for the multivariate Cox-PH model *P* value 1.15*e* − 07.

**Table 4:** CV-like performance of SVM classifier on training set and test of TCGA cohort.

Datasets	Omics type	Log-rank *P* value (geo.Mean)	C-index	Brier score
Training	3-omics	3.51*e* − 03	0.677 ± 0.042	0.216 ± 0.009

Test	3-omics	3.19*e* − 03	0.644 ± 0.030	0.209 ± 0.006
	mRNA only	3.56*e* − 03	0.636 ± 0.020	0.208 ± 0.008
	miRNA only	1.81*e* − 03	0.631 ± 0.017	0.201 ± 0.006
	Methylation only	2.63*e* − 03	0.653 ± 0.016	0.213 ± 0.004

## Data Availability

All data included in this study are available from The Cancer Genome Atlas (TCGA) database (http://cancergenome.nih.gov) and GEO repository (https://www.ncbi.nlm.nih.gov/geo/).

## Supplementary Materials

Supplementary Materials The following supplementary materials are available. Table S1: performance of models based on autoencoders with different numbers of nodes in hidden layers. Table S2: CV-like performance of models based on autoencoders with different numbers of nodes in hidden layers. Table S3: subgroup of patients from TCGA cohort. Table S4: top 50 mRNA, 30 miRNA, and 50 methylation features associated with subgroup based on ANOVA. Table S5: result of KEGG pathway enrichment analysis.
