# Supplementary material for: An Unsupervised Deep Learning-Based Model Using Multiomics Data to Predict Prognosis of Patients with Stomach Adenocarcinoma
Source: Comput Math Methods Med. 2022 Oct 27;2022:5844846. doi: 10.1155/2022/5844846 (PMC9633210; doi:10.1155/2022/5844846)
Supplement: Supplementary Materials — The following supplementary materials are available. Table S1: performance of models based on autoencoders with different numbers of nodes in hidden layers. Table S2: CV-like performance of models based on autoencoders with different numbers of nodes in hidden layers. Table S3: subgroup of patients from TCGA cohort. Table S4: top 50 mRNA, 30 miRNA, and 50 methylation features associated with subgroup based on ANOVA. Table S5: result of KEGG pathway enrichment analysis. [file 5844846.f1.docx]

Table S1 Performance of models based on autoencoders with different number of nodes in hidden layers

| number of nodes | log-rank P value | C-index | Brier Score |
| --- | --- | --- | --- |
| 1000 100 1000 | 1.81e-02 | 0.614 | 0.214 |
| 1000 200 1000 | 2.20e-03 | 0.658 | 0.216 |
| 1000 300 1000 | 1.20e-03 | 0.645 | 0.199 |
| 1000 500 1000 | 1.37e-04 | 0.669 | 0.209 |
| 1500 100 1500 | 6.56e-03 | 0.641 | 0.216 |
| 1500 200 1500 | 1.24e-02 | 0.622 | 0.213 |
| 1500 300 1500 | 1.25e-04 | 0.687 | 0.205 |
| 1500 500 1500 | 2.02e-05 | 0.697 | 0.210 |
| 2000 100 2000 | 1.60e-02 | 0.632 | 0.213 |
| 2000 200 2000 | 4.14e-03 | 0.658 | 0.207 |
| 2000 300 2000 | 2.21e-04 | 0.681 | 0.216 |
| 2000 500 2000 | 2.80e-06 | 0.714 | 0.184 |
| 3000 100 3000 | 1.03e-03 | 0.660 | 0.210 |
| 3000 200 3000 | 1.17e-03 | 0.651 | 0.209 |
| 3000 300 3000 | 2.81e-05 | 0.702 | 0.211 |
| 3000 500 3000 | 2.35e-05 | 0.691 | 0.201 |
| 5000 100 5000 | 2.35e-03 | 0.655 | 0.216 |
| 5000 200 5000 | 3.35e-04 | 0.657 | 0.210 |
| 5000 300 5000 | 1.04e-03 | 0.639 | 0.204 |
| 5000 500 5000 | 9.63e-08 | 0.741 | 0.206 |

Performance of models based on autoencoders with different number of nodes in hidden layers were shown in Table S1. Difference between high-risk and low-risk group were all significant in different autoencoders. The model based on autoencoder with a number of hidden layer nodes of 5000, 500, 5000 produced the lowest log-rank P value (9.63e-08) and the highest C-index (0.741). The model based on autoencoder with a number of hidden layer nodes of 2000, 500, 2000 produced the second lowest log-rank P value (2.80e-06), the second highest C-index (0.714) and the lowest brier score (0.184).

Table S2 CV-like performance of models based on autoencoders with different number of nodes in hidden layers

| number of nodes | datasets | omics type | Log-rank.P.value  (geo.mean) | C-index | Brier score |
| --- | --- | --- | --- | --- | --- |
| 1000 100 1000 | training | 3-omics | 3.51e-02 | 0.640±0.007 | 0.211±0.001 |
|  | test | 3-omics | 2.36e-02 | 0.620±0.008 | 0.214±0.001 |
|  |  | mRNA only | 5.43e-03 | 0.640±0.018 | 0.212±0.001 |
|  |  | miRNA only | 1.56e-02 | 0.611±0.026 | 0.212±0.002 |
|  |  | methylation only | 2.30e-02 | 0.619±0.006 | 0.213±0.001 |
| 1000 200 1000 | training | 3-omics | 2.91e-01 | 0.591±0.009 | 0.221±0.001 |
|  | test | 3-omics | 3.31e-02 | 0.621±0.008 | 0.208±0.001 |
|  |  | mRNA only | 1.68e-02 | 0.639±0.013 | 0.210±0.002 |
|  |  | miRNA only | 2.00e-02 | 0.644±0.029 | 0.207±0.001 |
|  |  | methylation only | 1.10e-01 | 0.593±0.014 | 0.210±0.001 |
| 1000 300 1000 | training | 3-omics | 1.77e-01 | 0.592±0.014 | 0.220±0.001 |
|  | test | 3-omics | 1.04e-01 | 0.589±0.021 | 0.216±0.002 |
|  |  | mRNA only | 1.49e-02 | 0.633±0.019 | 0.213±0.004 |
|  |  | miRNA only | 1.17e-01 | 0.571±0.022 | 0.209±0.002 |
|  |  | methylation only | 1.36e-01 | 0.575±0.015 | 0.215±0.002 |
| 1000 500 1000 | training | 3-omics | 1.93e-02 | 0.676±0.031 | 0.223±0.004 |
|  | test | 3-omics | 1.24e-03 | 0.675±0.013 | 0.213±0.002 |
|  |  | mRNA only | 4.79e-04 | 0.714±0.020 | 0.216±0.002 |
|  |  | miRNA only | 6.43e-03 | 0.644±0.038 | 0.205±0.008 |
|  |  | methylation only | 1.62e-02 | 0.636±0.019 | 0.222±0.004 |
| 1500 100 1500 | training | 3-omics | 1.30e-01 | 0.596±0.007 | 0.221±0.002 |
|  | test | 3-omics | 6.98e-03 | 0.631±0.006 | 0.212±0.002 |
|  |  | mRNA only | 2.26e-03 | 0.637±0.015 | 0.208±0.001 |
|  |  | miRNA only | 6.66e-03 | 0.619±0.010 | 0.205±0.002 |
|  |  | methylation only | 4.96e-02 | 0.600±0.009 | 0.210±0.001 |
| 1500 200 1500 | training | 3-omics | 1.94e-02 | 0.721±0.005 | 0.207±0.000 |
|  | test | 3-omics | 8.16e-02 | 0.631±0.012 | 0.218±0.001 |
|  |  | mRNA only | 1.90e-02 | 0.680±0.019 | 0.216±0.006 |
|  |  | miRNA only | 2.45e-02 | 0.719±0.018 | 0.214±0.001 |
|  |  | methylation only | 1.29e-01 | 0.598±0.018 | 0.219±0.002 |
| 1500 300 1500 | training | 3-omics | 1.08e-02 | 0.670±0.008 | 0.229±0.001 |
|  | test | 3-omics | 5.27e-03 | 0.646±0.005 | 0.219±0.001 |
|  |  | mRNA only | 8.48e-04 | 0.665±0.019 | 0.215±0.006 |
|  |  | miRNA only | 2.19e-03 | 0.652±0.011 | 0.211±0.004 |
|  |  | methylation only | 4.96e-02 | 0.605±0.004 | 0.217±0.001 |
| 1500 500 1500 | training | 3-omics | 1.32e-03 | 0.658±0.024 | 0.195±0.005 |
|  | test | 3-omics | 2.52e-02 | 0.589±0.020 | 0.207±0.007 |
|  |  | mRNA only | 4.95e-03 | 0.632±0.035 | 0.213±0.004 |
|  |  | miRNA only | 2.94e-02 | 0.587±0.013 | 0.208±0.006 |
|  |  | methylation only | 7.21e-02 | 0.581±0.015 | 0.212±0.008 |
| 2000 100 2000 | training | 3-omics | 2.91e-01 | 0.587±0.013 | 0.225±0.001 |
|  | test | 3-omics | 5.87e-03 | 0.641±0.009 | 0.211±0.001 |
|  |  | mRNA only | 1.45e-02 | 0.621±0.015 | 0.210±0.001 |
|  |  | miRNA only | 2.41e-03 | 0.652±0.019 | 0.210±0.001 |
|  |  | methylation only | 4.65e-02 | 0.609±0.008 | 0.212±0.001 |
| 2000 200 2000 | training | 3-omics | 2.55e-01 | 0.609±0.008 | 0.216±0.000 |
|  | test | 3-omics | 8.40e-02 | 0.640±0.008 | 0.210±0.000 |
|  |  | mRNA only | 3.28e-02 | 0.666±0.006 | 0.210±0.001 |
|  |  | miRNA only | 1.72e-02 | 0.792±0.015 | 0.207±0.000 |
|  |  | methylation only | 1.10e-01 | 0.624±0.022 | 0.210±0.002 |
| 2000 300 2000 | training | 3-omics | 1.53e-02 | 0.645±0.008 | 0.207±0.003 |
|  | test | 3-omics | 3.98e-03 | 0.647±0.013 | 0.218±0.002 |
|  |  | mRNA only | 2.02e-03 | 0.663±0.009 | 0.211±0.001 |
|  |  | miRNA only | 1.91e-02 | 0.623±0.006 | 0.211±0.002 |
|  |  | methylation only | 2.97e-02 | 0.614±0.009 | 0.218±0.002 |
| 2000 500 2000 | training | 3-omics | 3.51e-03 | 0.677±0.042 | 0.216±0.009 |
|  | test | 3-omics | 3.19e-03 | 0.644±0.030 | 0.209±0.006 |
|  |  | mRNA only | 3.56e-03 | 0.636±0.020 | 0.208±0.008 |
|  |  | miRNA only | 1.81e-03 | 0.631±0.017 | 0.201±0.006 |
|  |  | methylation only | 2.63e-03 | 0.653±0.016 | 0.213±0.004 |
| 3000 100 3000 | training | 3-omics | 1.78e-01 | 0.575±0.015 | 0.216±0.001 |
|  | test | 3-omics | 1.14e-03 | 0.634±0.011 | 0.207±0.002 |
|  |  | mRNA only | 2.78e-04 | 0.656±0.013 | 0.204±0.001 |
|  |  | miRNA only | 7.92e-04 | 0.650±0.013 | 0.207±0.001 |
|  |  | methylation only | 1.04e-02 | 0.615±0.019 | 0.212±0.001 |
| 3000 200 3000 | training | 3-omics | 7.80e-02 | 0.608±0.006 | 0.219±0.006 |
|  | test | 3-omics | 1.63e-03 | 0.647±0.007 | 0.203±0.003 |
|  |  | mRNA only | 5.66e-04 | 0.666±0.010 | 0.209±0.000 |
|  |  | miRNA only | 1.95e-03 | 0.644±0.013 | 0.200±0.002 |
|  |  | methylation only | 4.48e-02 | 0.617±0.016 | 0.211±0.001 |
| 3000 300 3000 | training | 3-omics | 5.24e-03 | 0.711±0.057 | 0.217±0.004 |
|  | test | 3-omics | 5.30e-03 | 0.672±0.023 | 0.220±0.009 |
|  |  | mRNA only | 8.55e-04 | 0.705±0.050 | 0.213±0.007 |
|  |  | miRNA only | 2.59e-03 | 0.680±0.094 | 0.213±0.004 |
|  |  | methylation only | 9.27e-03 | 0.662±0.038 | 0.218±0.010 |
| 3000 500 3000 | training | 3-omics | 5.34e-04 | 0.666±0.032 | 0.187±0.007 |
|  | test | 3-omics | 1.06e-03 | 0.646±0.025 | 0.213±0.009 |
|  |  | mRNA only | 9.35e-04 | 0.658±0.029 | 0.215±0.003 |
|  |  | miRNA only | 2.08e-02 | 0.604±0.020 | 0.215±0.005 |
|  |  | methylation only | 1.71e-02 | 0.607±0.046 | 0.228±0.013 |
| 5000 100 5000 | training | 3-omics | 3.83e-02 | 0.585±0.007 | 0.190±0.000 |
|  | test | 3-omics | 3.32e-01 | 0.559±0.009 | 0.225±0.002 |
|  |  | mRNA only | 1.46e-01 | 0.580±0.006 | 0.224±0.001 |
|  |  | miRNA only | 4.76e-02 | 0.592±0.012 | 0.215±0.004 |
|  |  | methylation only | 4.69e-01 | 0.547±0.008 | 0.225±0.002 |
| 5000 200 5000 | training | 3-omics | 1.18e-03 | 0.679±0.038 | 0.208±0.008 |
|  | test | 3-omics | 1.69e-02 | 0.616±0.039 | 0.205±0.003 |
|  |  | mRNA only | 7.98e-02 | 0.588±0.049 | 0.210±0.009 |
|  |  | miRNA only | 2.68e-02 | 0.607±0.031 | 0.206±0.004 |
|  |  | methylation only | 1.03e-01 | 0.589±0.018 | 0.211±0.011 |
| 5000 300 5000 | training | 3-omics | 4.77e-04 | 0.693±0.013 | 0.188±0.008 |
|  | test | 3-omics | 7.60e-04 | 0.648±0.016 | 0.212±0.013 |
|  |  | mRNA only | 1.88e-03 | 0.665±0.021 | 0.215±0.006 |
|  |  | miRNA only | 7.09e-05 | 0.710±0.025 | 0.195±0.006 |
|  |  | methylation only | 8.93e-02 | 0.561±0.020 | 0.222±0.009 |
| 5000 500 5000 | training | 3-omics | 2.56e-02 | 0.625±0.036 | 0.217±0.001 |
|  | test | 3-omics | 8.52e-02 | 0.577±0.022 | 0.210±0.002 |
|  |  | mRNA only | 5.37e-01 | 0.517±0.020 | 0.209±0.008 |
|  |  | miRNA only | 8.24e-01 | 0.497±0.010 | 0.213±0.006 |
|  |  | methylation only | 2.07e-01 | 0.560±0.018 | 0.214±0.003 |

CV-like performance of models based on autoencoders with different number of nodes in hidden layers were shown in Table S2.

The model based on autoencoder with a number of hidden layer nodes of 5000, 500, 5000 produced a bad performance in the CV-like procedure in test set. For SVM using 3-omic data, log-rank P value was not significant (8.52e-02) and low C-index was low (0.577±0.022). For SVM using mRNA data only, log-rank P value was not significant (5.37e-01) and low C-index was low (0.517±0.020). For SVM using miRNA data only, log-rank P value was not significant (8.24e-01) and low C-index was low (0.497±0.010). For SVM using methylation data only, log-rank P value was not significant (2.07e-01) and low C-index was low (0.560±0.018).

The model based on autoencoder with a number of hidden layer nodes of 2000, 500, 2000 produced an excellent performance in the CV-like procedure in both training set and test set. For training set, a high C-index of 0.677 ± 0.042, a low brier score of 0.216 ± 0.009 and a significant log-rank P value of 3.51e-03 were generated. For test set, a high C-index of 0.644 ± 0.030, a low brier score of 0.209 ± 0.006 and a significant log-rank P value of 3.19e-03 were generated (Table 3). SVMs using each three single omics features were also built and impressive performances were also produced. For test set based on mRNA features only, a C-index of 0.636 ± 0.020, a brier score of 0.208 ± 0.008 and a log-rank P value of 3.56e-03 were generated. For test set based on miRNA features only, a C-index of 0.631 ± 0.017, a brier score of 0.201 ± 0.006 and a log-rank P value of 1.81e-03 were generated. For test set based on methylation features only, a C-index of 0.653 ± 0.016, a brier score of 0.213 ± 0.004 and a log-rank P value of 2.63e-03 were generated.

Table S3 subgroup of patients from TCGA cohort

| sample | subgroup |
| --- | --- |
| TCGA-3M-AB46 | high-risk |
| TCGA-B7-5816 | high-risk |
| TCGA-B7-5818 | low-risk |
| TCGA-B7-A5TI | low-risk |
| TCGA-B7-A5TJ | high-risk |
| TCGA-B7-A5TK | low-risk |
| TCGA-B7-A5TN | high-risk |
| TCGA-BR-6452 | low-risk |
| TCGA-BR-6453 | high-risk |
| TCGA-BR-6454 | high-risk |
| TCGA-BR-6455 | high-risk |
| TCGA-BR-6456 | high-risk |
| TCGA-BR-6457 | high-risk |
| TCGA-BR-6458 | high-risk |
| TCGA-BR-6563 | high-risk |
| TCGA-BR-6564 | high-risk |
| TCGA-BR-6565 | high-risk |
| TCGA-BR-6566 | low-risk |
| TCGA-BR-6705 | high-risk |
| TCGA-BR-6706 | high-risk |
| TCGA-BR-6707 | low-risk |
| TCGA-BR-6709 | high-risk |
| TCGA-BR-6801 | high-risk |
| TCGA-BR-6802 | high-risk |
| TCGA-BR-6803 | high-risk |
| TCGA-BR-6852 | low-risk |
| TCGA-BR-7196 | low-risk |
| TCGA-BR-7197 | high-risk |
| TCGA-BR-7703 | low-risk |
| TCGA-BR-7704 | high-risk |
| TCGA-BR-7707 | low-risk |
| TCGA-BR-7715 | high-risk |
| TCGA-BR-7716 | high-risk |
| TCGA-BR-7717 | high-risk |
| TCGA-BR-7722 | high-risk |
| TCGA-BR-7723 | high-risk |
| TCGA-BR-7851 | low-risk |
| TCGA-BR-7901 | high-risk |
| TCGA-BR-7957 | high-risk |
| TCGA-BR-7958 | low-risk |
| TCGA-BR-7959 | high-risk |
| TCGA-BR-8058 | high-risk |
| TCGA-BR-8059 | high-risk |
| TCGA-BR-8060 | high-risk |
| TCGA-BR-8077 | high-risk |
| TCGA-BR-8078 | low-risk |
| TCGA-BR-8080 | high-risk |
| TCGA-BR-8081 | low-risk |
| TCGA-BR-8284 | low-risk |
| TCGA-BR-8286 | low-risk |
| TCGA-BR-8289 | high-risk |
| TCGA-BR-8291 | high-risk |
| TCGA-BR-8295 | high-risk |
| TCGA-BR-8296 | low-risk |
| TCGA-BR-8297 | high-risk |
| TCGA-BR-8361 | low-risk |
| TCGA-BR-8362 | high-risk |
| TCGA-BR-8363 | low-risk |
| TCGA-BR-8364 | high-risk |
| TCGA-BR-8365 | high-risk |
| TCGA-BR-8366 | low-risk |
| TCGA-BR-8367 | high-risk |
| TCGA-BR-8368 | low-risk |
| TCGA-BR-8369 | high-risk |
| TCGA-BR-8371 | high-risk |
| TCGA-BR-8372 | low-risk |
| TCGA-BR-8373 | high-risk |
| TCGA-BR-8380 | high-risk |
| TCGA-BR-8381 | low-risk |
| TCGA-BR-8382 | low-risk |
| TCGA-BR-8384 | high-risk |
| TCGA-BR-8483 | high-risk |
| TCGA-BR-8484 | low-risk |
| TCGA-BR-8485 | high-risk |
| TCGA-BR-8486 | high-risk |
| TCGA-BR-8487 | low-risk |
| TCGA-BR-8588 | low-risk |
| TCGA-BR-8589 | low-risk |
| TCGA-BR-8590 | high-risk |
| TCGA-BR-8591 | low-risk |
| TCGA-BR-8592 | high-risk |
| TCGA-BR-8676 | low-risk |
| TCGA-BR-8677 | high-risk |
| TCGA-BR-8678 | high-risk |
| TCGA-BR-8679 | low-risk |
| TCGA-BR-8680 | low-risk |
| TCGA-BR-8682 | high-risk |
| TCGA-BR-8683 | high-risk |
| TCGA-BR-8686 | low-risk |
| TCGA-BR-8687 | high-risk |
| TCGA-BR-8690 | high-risk |
| TCGA-BR-A44T | high-risk |
| TCGA-BR-A44U | low-risk |
| TCGA-BR-A452 | high-risk |
| TCGA-BR-A453 | high-risk |
| TCGA-BR-A4CR | high-risk |
| TCGA-BR-A4CS | high-risk |
| TCGA-BR-A4IU | high-risk |
| TCGA-BR-A4IV | high-risk |
| TCGA-BR-A4IY | low-risk |
| TCGA-BR-A4IZ | high-risk |
| TCGA-BR-A4J1 | high-risk |
| TCGA-BR-A4J2 | high-risk |
| TCGA-BR-A4J4 | low-risk |
| TCGA-BR-A4J5 | high-risk |
| TCGA-BR-A4J6 | low-risk |
| TCGA-BR-A4J7 | high-risk |
| TCGA-BR-A4J8 | high-risk |
| TCGA-BR-A4J9 | high-risk |
| TCGA-BR-A4PD | high-risk |
| TCGA-BR-A4PE | low-risk |
| TCGA-BR-A4PF | low-risk |
| TCGA-BR-A4QI | low-risk |
| TCGA-BR-A4QL | low-risk |
| TCGA-BR-A4QM | high-risk |
| TCGA-CD-5798 | high-risk |
| TCGA-CD-5799 | high-risk |
| TCGA-CD-5800 | high-risk |
| TCGA-CD-5801 | high-risk |
| TCGA-CD-5803 | high-risk |
| TCGA-CD-5804 | high-risk |
| TCGA-CD-5813 | high-risk |
| TCGA-CD-8524 | high-risk |
| TCGA-CD-8525 | high-risk |
| TCGA-CD-8526 | high-risk |
| TCGA-CD-8527 | high-risk |
| TCGA-CD-8528 | low-risk |
| TCGA-CD-8529 | high-risk |
| TCGA-CD-8530 | high-risk |
| TCGA-CD-8531 | low-risk |
| TCGA-CD-8532 | high-risk |
| TCGA-CD-8533 | high-risk |
| TCGA-CD-8534 | low-risk |
| TCGA-CD-8535 | high-risk |
| TCGA-CD-8536 | low-risk |
| TCGA-CD-A486 | high-risk |
| TCGA-CD-A487 | high-risk |
| TCGA-CD-A489 | high-risk |
| TCGA-CD-A48A | low-risk |
| TCGA-CD-A48C | high-risk |
| TCGA-CD-A4MG | low-risk |
| TCGA-CD-A4MH | high-risk |
| TCGA-CD-A4MI | low-risk |
| TCGA-CD-A4MJ | low-risk |
| TCGA-CG-4437 | low-risk |
| TCGA-CG-4441 | high-risk |
| TCGA-CG-5717 | high-risk |
| TCGA-CG-5718 | high-risk |
| TCGA-CG-5719 | high-risk |
| TCGA-CG-5720 | high-risk |
| TCGA-CG-5721 | high-risk |
| TCGA-CG-5722 | high-risk |
| TCGA-CG-5723 | high-risk |
| TCGA-CG-5724 | high-risk |
| TCGA-CG-5725 | high-risk |
| TCGA-CG-5726 | high-risk |
| TCGA-CG-5732 | high-risk |
| TCGA-CG-5734 | high-risk |
| TCGA-D7-5577 | low-risk |
| TCGA-D7-5578 | high-risk |
| TCGA-D7-6518 | high-risk |
| TCGA-D7-6519 | high-risk |
| TCGA-D7-6520 | high-risk |
| TCGA-D7-6521 | high-risk |
| TCGA-D7-6522 | high-risk |
| TCGA-D7-6524 | high-risk |
| TCGA-D7-6525 | high-risk |
| TCGA-D7-6526 | high-risk |
| TCGA-D7-6527 | high-risk |
| TCGA-D7-6528 | high-risk |
| TCGA-D7-6815 | high-risk |
| TCGA-D7-6817 | high-risk |
| TCGA-D7-6818 | high-risk |
| TCGA-D7-6820 | high-risk |
| TCGA-D7-6822 | high-risk |
| TCGA-D7-8570 | low-risk |
| TCGA-D7-8572 | high-risk |
| TCGA-D7-8573 | low-risk |
| TCGA-D7-8574 | high-risk |
| TCGA-D7-8575 | low-risk |
| TCGA-D7-8576 | low-risk |
| TCGA-D7-8578 | high-risk |
| TCGA-D7-8579 | high-risk |
| TCGA-D7-A4YT | low-risk |
| TCGA-D7-A4YU | low-risk |
| TCGA-D7-A4YV | low-risk |
| TCGA-D7-A4YX | low-risk |
| TCGA-D7-A4YY | low-risk |
| TCGA-D7-A4Z0 | high-risk |
| TCGA-D7-A6EV | low-risk |
| TCGA-D7-A6EX | high-risk |
| TCGA-D7-A6EY | low-risk |
| TCGA-D7-A6EZ | low-risk |
| TCGA-D7-A6F0 | high-risk |
| TCGA-D7-A6F2 | high-risk |
| TCGA-D7-A747 | high-risk |
| TCGA-D7-A748 | high-risk |
| TCGA-D7-A74A | low-risk |
| TCGA-EQ-8122 | high-risk |
| TCGA-EQ-A4SO | low-risk |
| TCGA-F1-6177 | low-risk |
| TCGA-F1-6874 | low-risk |
| TCGA-F1-6875 | high-risk |
| TCGA-F1-A448 | high-risk |
| TCGA-F1-A72C | high-risk |
| TCGA-FP-7735 | high-risk |
| TCGA-FP-7829 | high-risk |
| TCGA-FP-7916 | low-risk |
| TCGA-FP-7998 | low-risk |
| TCGA-FP-8099 | low-risk |
| TCGA-FP-8209 | high-risk |
| TCGA-FP-8210 | high-risk |
| TCGA-FP-8211 | high-risk |
| TCGA-FP-8631 | high-risk |
| TCGA-FP-A4BE | low-risk |
| TCGA-FP-A4BF | high-risk |
| TCGA-FP-A8CX | low-risk |
| TCGA-FP-A9TM | low-risk |
| TCGA-HF-7132 | low-risk |
| TCGA-HF-7133 | low-risk |
| TCGA-HF-7134 | low-risk |
| TCGA-HF-A5NB | low-risk |
| TCGA-HJ-7597 | low-risk |
| TCGA-HU-8238 | high-risk |
| TCGA-HU-8243 | high-risk |
| TCGA-HU-8244 | low-risk |
| TCGA-HU-8249 | low-risk |
| TCGA-HU-8602 | low-risk |
| TCGA-HU-8604 | low-risk |
| TCGA-HU-8608 | low-risk |
| TCGA-HU-8610 | high-risk |
| TCGA-HU-A4G2 | low-risk |
| TCGA-HU-A4G3 | high-risk |
| TCGA-HU-A4G6 | low-risk |
| TCGA-HU-A4G8 | low-risk |
| TCGA-HU-A4G9 | low-risk |
| TCGA-HU-A4GC | high-risk |
| TCGA-HU-A4GD | high-risk |
| TCGA-HU-A4GF | high-risk |
| TCGA-HU-A4GH | high-risk |
| TCGA-HU-A4GJ | high-risk |
| TCGA-HU-A4GN | low-risk |
| TCGA-HU-A4GP | high-risk |
| TCGA-HU-A4GQ | high-risk |
| TCGA-HU-A4GT | low-risk |
| TCGA-HU-A4GU | low-risk |
| TCGA-HU-A4GX | low-risk |
| TCGA-HU-A4GY | high-risk |
| TCGA-HU-A4H0 | low-risk |
| TCGA-HU-A4H2 | low-risk |
| TCGA-HU-A4H3 | low-risk |
| TCGA-HU-A4H4 | low-risk |
| TCGA-HU-A4H5 | high-risk |
| TCGA-HU-A4H6 | low-risk |
| TCGA-HU-A4HB | high-risk |
| TCGA-HU-A4HD | high-risk |
| TCGA-IN-7806 | high-risk |
| TCGA-IN-7808 | high-risk |
| TCGA-IN-8462 | high-risk |
| TCGA-IN-8663 | low-risk |
| TCGA-IN-A6RI | high-risk |
| TCGA-IN-A6RJ | high-risk |
| TCGA-IN-A6RL | high-risk |
| TCGA-IN-A6RN | high-risk |
| TCGA-IN-A6RR | low-risk |
| TCGA-IN-A6RS | low-risk |
| TCGA-IN-A7NR | high-risk |
| TCGA-IN-A7NT | high-risk |
| TCGA-IN-A7NU | high-risk |
| TCGA-IN-AB1V | high-risk |
| TCGA-IN-AB1X | low-risk |
| TCGA-IP-7968 | high-risk |
| TCGA-KB-A6F7 | low-risk |
| TCGA-KB-A93J | high-risk |
| TCGA-MX-A5UG | high-risk |
| TCGA-MX-A5UJ | low-risk |
| TCGA-MX-A663 | high-risk |
| TCGA-MX-A666 | high-risk |
| TCGA-R5-A7O7 | high-risk |
| TCGA-R5-A7ZE | high-risk |
| TCGA-R5-A7ZF | high-risk |
| TCGA-R5-A7ZI | low-risk |
| TCGA-R5-A7ZR | high-risk |
| TCGA-R5-A805 | low-risk |
| TCGA-RD-A7BS | high-risk |
| TCGA-RD-A7BW | high-risk |
| TCGA-RD-A7C1 | high-risk |
| TCGA-RD-A8MV | low-risk |
| TCGA-RD-A8MW | high-risk |
| TCGA-RD-A8N0 | high-risk |
| TCGA-RD-A8N1 | low-risk |
| TCGA-RD-A8N2 | high-risk |
| TCGA-RD-A8N4 | high-risk |
| TCGA-RD-A8N5 | high-risk |
| TCGA-RD-A8N6 | high-risk |
| TCGA-RD-A8N9 | high-risk |
| TCGA-RD-A8NB | high-risk |
| TCGA-SW-A7EA | low-risk |
| TCGA-SW-A7EB | high-risk |
| TCGA-VQ-A8DT | low-risk |
| TCGA-VQ-A8DU | high-risk |
| TCGA-VQ-A8DV | high-risk |
| TCGA-VQ-A8DZ | high-risk |
| TCGA-VQ-A8E0 | low-risk |
| TCGA-VQ-A8E2 | high-risk |
| TCGA-VQ-A8E3 | low-risk |
| TCGA-VQ-A8E7 | high-risk |
| TCGA-VQ-A8P2 | low-risk |
| TCGA-VQ-A8P3 | low-risk |
| TCGA-VQ-A8P5 | high-risk |
| TCGA-VQ-A8P8 | low-risk |
| TCGA-VQ-A8PB | low-risk |
| TCGA-VQ-A8PC | high-risk |
| TCGA-VQ-A8PD | high-risk |
| TCGA-VQ-A8PE | high-risk |
| TCGA-VQ-A8PF | low-risk |
| TCGA-VQ-A8PH | high-risk |
| TCGA-VQ-A8PJ | high-risk |
| TCGA-VQ-A8PK | high-risk |
| TCGA-VQ-A8PM | high-risk |
| TCGA-VQ-A8PO | low-risk |
| TCGA-VQ-A8PP | high-risk |
| TCGA-VQ-A8PQ | high-risk |
| TCGA-VQ-A8PU | low-risk |
| TCGA-VQ-A8PX | low-risk |
| TCGA-VQ-A91A | high-risk |
| TCGA-VQ-A91D | low-risk |
| TCGA-VQ-A91E | low-risk |
| TCGA-VQ-A91K | low-risk |
| TCGA-VQ-A91N | high-risk |
| TCGA-VQ-A91Q | high-risk |
| TCGA-VQ-A91S | low-risk |
| TCGA-VQ-A91U | high-risk |
| TCGA-VQ-A91V | low-risk |
| TCGA-VQ-A91W | low-risk |
| TCGA-VQ-A91X | high-risk |
| TCGA-VQ-A91Y | high-risk |
| TCGA-VQ-A91Z | low-risk |
| TCGA-VQ-A922 | high-risk |
| TCGA-VQ-A923 | low-risk |
| TCGA-VQ-A924 | low-risk |
| TCGA-VQ-A925 | high-risk |
| TCGA-VQ-A927 | high-risk |
| TCGA-VQ-A928 | high-risk |
| TCGA-VQ-A92D | low-risk |
| TCGA-VQ-A94O | high-risk |
| TCGA-VQ-A94P | high-risk |
| TCGA-VQ-A94R | high-risk |
| TCGA-VQ-A94T | low-risk |
| TCGA-VQ-A94U | low-risk |
| TCGA-VQ-AA64 | high-risk |
| TCGA-VQ-AA68 | low-risk |
| TCGA-VQ-AA69 | low-risk |
| TCGA-VQ-AA6A | high-risk |
| TCGA-VQ-AA6B | low-risk |
| TCGA-VQ-AA6D | high-risk |
| TCGA-VQ-AA6F | low-risk |
| TCGA-VQ-AA6G | low-risk |
| TCGA-VQ-AA6I | low-risk |
| TCGA-VQ-AA6J | high-risk |
| TCGA-VQ-AA6K | high-risk |
| TCGA-ZA-A8F6 | high-risk |
| TCGA-ZQ-A9CR | high-risk |

The subgroups f patients from TCGA cohort obtained from the model based on autoencoder were shown in Table S3.

Table S4 Top 50 mRNA, 30 miRNA and 50 methylation features associated with subgroup based on ANOVA

| mRNA | P value | miRNA | P value | Methylation | P value |
| --- | --- | --- | --- | --- | --- |
| ETNK2 | 2.44E-35 | hsa-mir-497 | 2.63E-19 | EPO | 3.41E-43 |
| LARP6 | 2.37E-33 | hsa-mir-99b | 9.76E-19 | BDNF | 3.20E-40 |
| APBB1 | 6.42E-33 | hsa-mir-337 | 1.81E-18 | LHX4 | 7.02E-38 |
| USP2 | 6.98E-31 | hsa-mir-125a | 3.13E-17 | RRP15 | 6.03E-37 |
| PIFO | 1.22E-30 | hsa-mir-625 | 6.12E-16 | INSRR | 1.16E-36 |
| GNPNAT1 | 2.16E-29 | hsa-mir-218-2 | 3.53E-15 | ADAM11 | 1.63E-36 |
| FAM120A | 3.04E-29 | hsa-mir-195 | 3.72E-15 | NPAS1 | 4.60E-35 |
| TMEM161B | 3.46E-29 | hsa-mir-130a | 7.10E-15 | BAIAP3 | 7.79E-35 |
| CTSF | 8.31E-29 | hsa-mir-654 | 1.75E-14 | LHX9 | 8.14E-35 |
| PALM | 3.13E-28 | hsa-mir-15a | 7.34E-14 | ISLR2 | 8.96E-35 |
| SHF | 6.09E-28 | hsa-mir-125b-1 | 1.29E-13 | LOC283731 | 8.96E-35 |
| TCF7L1 | 1.51E-27 | hsa-mir-185 | 1.33E-13 | MYH11 | 1.90E-34 |
| PPP1R14A | 2.73E-27 | hsa-mir-187 | 3.97E-13 | CYB5R1 | 7.87E-34 |
| CERS4 | 4.28E-27 | hsa-mir-99a | 9.87E-13 | TMEM200B | 8.51E-34 |
| PDLIM4 | 4.51E-27 | hsa-mir-487b | 1.25E-12 | ATP1B2 | 9.03E-34 |
| MCCC2 | 6.16E-27 | hsa-mir-214 | 3.11E-12 | AMER3 | 1.03E-33 |
| PI4K2B | 7.95E-27 | hsa-mir-758 | 6.41E-12 | PDE9A | 1.33E-33 |
| SMO | 8.57E-27 | hsa-mir-200b | 6.83E-12 | SLC30A3 | 1.73E-33 |
| FBXO17 | 1.46E-26 | hsa-mir-182 | 8.88E-12 | MAD2L2 | 2.28E-33 |
| NPTXR | 1.62E-26 | hsa-mir-96 | 1.07E-11 | DRAXIN | 3.24E-33 |
| RGN | 1.71E-26 | hsa-mir-145 | 2.03E-11 | RIMKLB | 1.10E-32 |
| CNOT6 | 1.99E-26 | hsa-mir-125b-2 | 2.21E-11 | ELMOD1 | 1.27E-32 |
| LTBP3 | 2.05E-26 | hsa-mir-17 | 2.33E-11 | LOC643923 | 1.27E-32 |
| DHX15 | 2.59E-26 | hsa-mir-942 | 2.40E-11 | HRK | 1.66E-32 |
| MARK1 | 2.89E-26 | hsa-mir-20a | 2.71E-11 | NPY1R | 2.10E-32 |
| TLCD5 | 3.38E-26 | hsa-mir-4662a | 3.29E-11 | PAX2 | 2.39E-32 |
| UTP15 | 3.89E-26 | hsa-mir-29b-1 | 9.98E-11 | COL9A3 | 2.40E-32 |
| LOC149837 | 4.01E-26 | hsa-mir-107 | 9.99E-11 | RSPO4 | 2.47E-32 |
| RND2 | 5.63E-26 | hsa-mir-93 | 1.01E-10 | FHOD3 | 3.71E-32 |
| CLDN11 | 5.96E-26 | hsa-mir-935 | 1.34E-10 | SHISA7 | 4.08E-32 |
| SLC22A17 | 1.36E-25 |  |  | FBXL15 | 5.20E-32 |
| SGCE | 1.42E-25 |  |  | PSD | 5.20E-32 |
| PDE9A | 1.86E-25 |  |  | UNCX | 5.29E-32 |
| FBLN1 | 2.65E-25 |  |  | CAMK2N2 | 9.23E-32 |
| SMIM15 | 3.23E-25 |  |  | TNFRSF1B | 9.52E-32 |
| RTL8B | 3.67E-25 |  |  | RGL3 | 1.12E-31 |
| AP3B1 | 5.29E-25 |  |  | KIF1A | 1.12E-31 |
| SALL2 | 7.76E-25 |  |  | FAM163A | 1.22E-31 |
| SV2A | 1.18E-24 |  |  | INSYN1 | 1.52E-31 |
| SLF1 | 1.34E-24 |  |  | RCL1 | 1.83E-31 |
| MRPS27 | 1.60E-24 |  |  | CBX7 | 2.22E-31 |
| ANKRD6 | 1.70E-24 |  |  | S1PR1 | 2.59E-31 |
| EXOC6 | 1.88E-24 |  |  | KCNA2 | 2.91E-31 |
| SCARA3 | 2.11E-24 |  |  | TRIM9 | 3.54E-31 |
| CACNG4 | 2.61E-24 |  |  | VASH1 | 3.78E-31 |
| GFRA3 | 2.94E-24 |  |  | PDGFRA | 4.26E-31 |
| RARS1 | 2.94E-24 |  |  | BRINP3 | 4.63E-31 |
| SHISA4 | 3.21E-24 |  |  | NRROS | 6.07E-31 |
| UHRF1 | 3.26E-24 |  |  | B3GAT1 | 7.95E-31 |
| ZNF853 | 3.43E-24 |  |  | LOC731779 | 1.02E-30 |

The top 50 miRNA, 30 miRNA and 50 methylation features associated with subgroup based on ANOVA were shown in Table S4 and then were used to construct a SVM classifier.

Table S5 Result of KEGG pathway enrichment analysis

| regulation | ID | Description | GeneRatio | BgRatio | p.adjust | qvalue | geneID | Count | Order |
| --- | --- | --- | --- | --- | --- | --- | --- | --- | --- |
| up | hsa04020 | Calcium signaling pathway | 60/711 | 240/8150 | 6.22E-12 | 5.41E-12 | 108/148/147/146/155/185/491/493/623/774/775/777/8913/8912/8911/815/816/844/845/1129/1131/1133/2066/2255/9965/2247/2250/2252/2260/2668/2774/2903/2925/3270/3356/55283/4638/91807/4803/4915/4916/4923/9127/5136/5153/5137/5260/5350/5731/5733/5737/5979/6261/6263/6543/6547/6869/6865/7125/2277 | 60 | Calcium signaling pathway |
|  | hsa04080 | Neuroactive ligand-receptor interaction | 68/711 | 362/8150 | 8.52E-08 | 7.42E-08 | 116/148/147/146/152/155/183/185/554/623/1129/1131/1132/1133/1136/1141/1143/1268/1269/1511/1813/9568/2564/2690/51738/2740/9340/2743/165829/2892/2894/2898/2899/2900/2901/2903/2914/2925/3356/3953/23566/66004/10316/283869/4886/4922/4923/5020/9127/9934/5179/5443/5644/5731/5733/5737/5745/1903/6344/6750/6752/6866/6869/6865/2837/7425/7432/7434 | 68 | Neuroactive ligand-receptor interaction |
|  | hsa05410 | Hypertrophic cardiomyopathy | 28/711 | 90/8150 | 9.09E-08 | 7.91E-08 | 70/183/775/781/9254/55799/783/27092/1674/1756/3479/3569/3674/3678/3679/8516/3680/284217/3908/5563/6442/6444/6543/6547/7042/7043/7168/7169 | 28 | Hypertrophic cardiomyopathy |
|  | hsa05414 | Dilated cardiomyopathy | 29/711 | 96/8150 | 9.09E-08 | 7.91E-08 | 70/108/111/183/775/781/9254/55799/783/27092/1674/1756/3479/3674/3678/3679/8516/3680/284217/3908/5350/6442/6444/6543/6547/7042/7043/7168/7169 | 29 | Dilated cardiomyopathy |
|  | hsa04514 | Cell adhesion molecules | 36/711 | 149/8150 | 6.37E-07 | 5.54E-07 | 23705/57863/1013/1000/1002/9071/5010/149461/7122/9074/9080/1272/6900/8506/152404/8516/3680/58494/83700/3897/64101/94030/57689/8174/4359/4684/4685/257194/23114/57502/4897/9378/9379/9369/22854/6403 | 36 | Cell adhesion molecules |
|  | hsa04512 | ECM-receptor interaction | 25/711 | 88/8150 | 3.33E-06 | 2.90E-06 | 948/1101/1285/1287/1288/1297/1299/1311/80144/158326/3674/3678/3679/8516/3680/284217/3908/5649/9900/9899/7060/3371/63923/7148/7448 | 25 | ECM-receptor interaction |
|  | hsa05412 | Arrhythmogenic right ventricular cardiomyopathy | 23/711 | 77/8150 | 3.46E-06 | 3.01E-06 | 88/775/781/9254/55799/783/27092/1000/29119/1674/1756/3674/3678/3679/8516/3680/284217/3908/6442/6444/6543/6547/83439 | 23 | Arrhythmogenic right ventricular cardiomyopathy |
|  | hsa04261 | Adrenergic signaling in cardiomyocytes | 34/711 | 150/8150 | 5.49E-06 | 4.78E-06 | 70/108/111/148/147/146/183/185/10000/477/482/491/493/775/781/9254/55799/783/27092/815/816/84699/486/4635/5350/5502/5522/6330/6331/6332/6543/6547/7168/7169 | 34 | Adrenergic signaling in cardiomyocytes |
|  | hsa04974 | Protein digestion and absorption | 25/711 | 103/8150 | 5.85E-05 | 5.09E-05 | 477/482/1302/7373/1310/81578/91522/84570/136227/1285/1287/1288/1295/1297/1299/2006/486/4225/4311/5644/6564/11136/6543/6547/7512 | 25 | Protein digestion and absorption |
|  | hsa04360 | Axon guidance | 35/711 | 182/8150 | 1.63E-04 | 1.42E-04 | 655/91653/815/816/1073/6387/1949/2042/2044/2045/2051/151449/3897/64101/57689/10398/4776/9423/4917/22854/5063/91584/56963/6092/10371/223117/9723/54437/10500/6585/9353/6586/6608/7220/7223 | 35 | Axon guidance |
|  | hsa04024 | cAMP signaling pathway | 40/711 | 221/8150 | 1.63E-04 | 1.42E-04 | 108/111/116/10000/477/482/491/493/775/815/816/1129/1261/84699/1813/5348/486/9568/51738/2735/2737/2740/2892/2903/610/10021/64399/5602/10398/4881/4886/5020/5139/5350/5443/5733/6750/6752/7432/7434 | 40 | cAMP signaling pathway |
|  | hsa04310 | Wnt signaling pathway | 33/711 | 170/8150 | 2.16E-04 | 1.88E-04 | 147495/164284/815/816/1501/80319/23500/2487/8324/8326/5602/4776/85407/85409/147111/144165/166336/4920/284654/340419/84870/343637/5176/6422/6423/6424/83439/57216/7481/7482/7475/7483/7484 | 33 | Wnt signaling pathway |
|  | hsa04925 | Aldosterone synthesis and secretion | 22/711 | 98/8150 | 6.36E-04 | 5.54E-04 | 108/111/183/185/477/482/491/493/775/8913/8912/8911/815/816/84699/1583/1589/3777/4881/5138/5443/5587 | 22 | Aldosterone synthesis and secretion |
|  | hsa04911 | Insulin secretion | 20/711 | 86/8150 | 7.94E-04 | 6.91E-04 | 108/111/116/477/482/775/815/816/1131/84699/486/2645/2740/3778/3779/10242/3780/27445/9699/6616 | 20 | Insulin secretion |
|  | hsa04270 | Vascular smooth muscle contraction | 26/711 | 134/8150 | 1.56E-03 | 1.36E-03 | 59/72/108/111/148/147/146/183/185/775/800/3778/3779/10242/4628/4629/10398/4638/91807/4881/64600/8605/5322/4660/94274/5592 | 26 | Vascular smooth muscle contraction |
|  | hsa04022 | cGMP-PKG signaling pathway | 30/711 | 167/8150 | 1.89E-03 | 1.64E-03 | 108/111/148/147/146/152/155/185/10000/477/482/491/493/775/84699/486/3778/3779/10242/10398/4638/91807/4776/4881/5138/5139/5350/5592/6543/6547 | 30 | cGMP-PKG signaling pathway |
|  | hsa04927 | Cortisol synthesis and secretion | 16/711 | 65/8150 | 1.89E-03 | 1.65E-03 | 108/111/183/185/775/8913/8912/8911/84699/1583/1589/3776/3777/5087/8622/5443 | 16 | Cortisol synthesis and secretion |
|  | hsa04510 | Focal adhesion | 34/711 | 201/8150 | 2.04E-03 | 1.78E-03 | 10000/857/1101/1285/1287/1288/1297/1299/1311/2316/2318/3479/3674/3678/3679/8516/3680/284217/3908/5602/10398/4638/91807/5063/4660/5923/5649/399694/7060/3371/63923/7148/2277/7448 | 34 | Focal adhesion |
|  | hsa04934 | Cushing syndrome | 28/711 | 155/8150 | 2.43E-03 | 2.12E-03 | 108/111/183/185/775/8913/8912/8911/815/816/898/1029/84699/1583/1589/8324/8326/3776/3777/5087/8622/5443/83439/7481/7482/7475/7483/7484 | 28 | Cushing syndrome |
|  | hsa04151 | PI3K-Akt signaling pathway | 50/711 | 354/8150 | 6.02E-03 | 5.24E-03 | 10000/284/898/930/1101/1129/1285/1287/1288/1297/1299/1311/84699/1440/2066/2069/2255/9965/2247/2250/2252/2260/2690/2786/3479/3481/3569/3674/3678/3679/8516/3680/284217/3908/23566/9863/4803/4804/4908/4915/5522/5563/5649/8115/7060/3371/63923/7148/2277/7448 | 50 | PI3K-Akt signaling pathway |
|  | hsa04260 | Cardiac muscle contraction | 18/711 | 87/8150 | 6.14E-03 | 5.35E-03 | 70/477/482/775/781/9254/55799/783/27092/845/1346/486/3270/4635/6543/6547/7168/7169 | 18 | Cardiac muscle contraction |
|  | hsa04713 | Circadian entrainment | 18/711 | 97/8150 | 2.15E-02 | 1.87E-02 | 108/111/116/775/8913/8912/8911/815/816/2775/2786/2892/2903/3763/3765/5592/6261/6263 | 18 | Circadian entrainment |
|  | hsa04010 | MAPK signaling pathway | 41/711 | 294/8150 | 2.15E-02 | 1.87E-02 | 10000/284/774/775/777/8913/8912/8911/781/9254/55799/783/27092/1852/2066/2069/2255/9965/2247/2250/2252/2260/2316/2318/3306/3479/3481/5602/9479/4137/4803/4804/4908/4915/8605/84867/5923/10235/7042/7043/2277 | 41 | MAPK signaling pathway |
|  | hsa04921 | Oxytocin signaling pathway | 25/711 | 154/8150 | 2.15E-02 | 1.87E-02 | 108/111/775/781/9254/55799/783/27092/815/816/2775/3768/3763/3765/10398/4638/91807/4776/4881/5020/8605/4660/5563/6261/6263 | 25 | Oxytocin signaling pathway |
|  | hsa04924 | Renin secretion | 14/711 | 69/8150 | 2.65E-02 | 2.30E-02 | 111/116/155/183/185/775/9635/22802/3778/4881/5136/5153/5137/5139 | 14 | Renin secretion |
|  | hsa04970 | Salivary secretion | 17/711 | 92/8150 | 2.65E-02 | 2.30E-02 | 108/111/148/147/146/155/477/482/491/493/1131/1470/1472/486/3778/5592/6263 | 17 | Salivary secretion |
|  | hsa05217 | Basal cell carcinoma | 13/711 | 63/8150 | 2.99E-02 | 2.60E-02 | 8324/8326/2735/2737/64399/8643/6608/83439/7481/7482/7475/7483/7484 | 13 | Basal cell carcinoma |
|  | hsa04340 | Hedgehog signaling pathway | 12/711 | 56/8150 | 2.99E-02 | 2.60E-02 | 91653/50937/50846/2121/132884/2619/2735/2737/64399/8643/57758/6608 | 12 | Hedgehog signaling pathway |
|  | hsa04972 | Pancreatic secretion | 18/711 | 102/8150 | 3.03E-02 | 2.64E-02 | 108/111/477/482/491/493/1056/1131/9635/22802/486/3778/64600/5322/5644/6344/1811/7220 | 18 | Pancreatic secretion |
|  | hsa04725 | Cholinergic synapse | 19/711 | 113/8150 | 3.99E-02 | 3.47E-02 | 108/111/10000/774/775/815/816/1129/1131/1132/1133/1136/1141/1143/84699/2775/2786/3768/3763 | 19 | Cholinergic synapse |
|  | hsa04724 | Glutamatergic synapse | 19/711 | 114/8150 | 4.26E-02 | 3.71E-02 | 108/111/775/1742/9229/2775/2786/2892/2898/2899/2900/2901/2903/2914/8605/50944/57030/10991/7220 | 19 | Glutamatergic synapse |
|  | hsa02010 | ABC transporters | 10/711 | 45/8150 | 4.39E-02 | 3.82E-02 | 23460/10351/10350/5244/340273/10060/9429/64137/64241/1672 | 10 | ABC transporters |
|  | hsa04978 | Mineral absorption | 12/711 | 60/8150 | 4.49E-02 | 3.91E-02 | 477/482/491/493/79901/486/341208/1811/6543/6547/7018/140803 | 12 | Mineral absorption |
|  | hsa05032 | Morphine addiction | 16/711 | 91/8150 | 4.49E-02 | 3.91E-02 | 108/111/774/9568/2564/2775/2786/3763/3765/5136/5153/5137/5138/5139/27115/8622 | 16 | Morphine addiction |
| down | hsa04612 | Antigen processing and presentation | 4/31 | 78/8150 | 2.07E-02 | 1.73E-02 | 3458/3805/3821/3822 | 4 | Antigen processing and presentation |
|  | hsa05332 | Graft-versus-host disease | 3/31 | 42/8150 | 2.51E-02 | 2.11E-02 | 3002/3458/3821 | 3 | Graft-versus-host disease |
|  | hsa04060 | Cytokine-cytokine receptor interaction | 6/31 | 295/8150 | 2.51E-02 | 2.11E-02 | 268/3627/6373/4283/3458/8744 | 6 | Cytokine-cytokine receptor interaction |
|  | hsa04650 | Natural killer cell mediated cytotoxicity | 4/31 | 131/8150 | 3.69E-02 | 3.09E-02 | 3002/3458/3821/3822 | 4 | Natural killer cell mediated cytotoxicity |

The result of KEGG pathway enrichment analysis were shown in Table S5. The upregulated and downregulated DEGs were significantly involved in 34 and 4 signaling pathways, respectively.
